# The Impact of Post-stroke Depression and Physical Fatigue on Functional Status

**DOI:** 10.62641/aep.v53i2.1688

**Published:** 2025-03-05

**Authors:** Fengying Hu, Kun Zhang, Liheng Zhou, Yanmei Wang

**Affiliations:** ^1^Department of Neurology, Taihe County People’s Hospital, 236600 Fuyang, Anhui, China

**Keywords:** post-stroke, depression, physical fatigue, functional status

## Abstract

**Background:**

Stroke is a leading cause of long-term disability globally, with post-stroke depression and physical fatigue recognized as prominent complications affecting recovery and rehabilitation. This study aims to comprehensively investigate the impact of post-stroke depression and physical fatigue on the functional outcomes of individuals who have experienced stroke.

**Methods:**

This research involved a retrospective analysis of clinical data from patients with stroke admitted to Taihe County People’s Hospital between January 2022 and May 2023. Patients were categorized into two groups based on their prognostic functional status: good and poor. The impact of post-stroke depression and physical fatigue on functional outcomes was assessed using standardized assessment tools. Specifically, the Patient Health Questionnaire-9 (PHQ-9) was employed to measure depression severity, while the Fatigue Severity Scale (FSS) was utilized to quantify physical fatigue.

**Results:**

Post-stroke depression and physical fatigue were significantly associated with functional status. The post-stroke depression scores were notably higher in the poor functional status group (10.58 ± 3.82) compared to the good functional status group (7.81 ± 2.12) (*t *= 4.482, *p* < 0.001). Similarly, post-stroke physical fatigue scores were significantly elevated in the poor functional status group (56.87 ± 2.53) compared to the good functional status group (43.26 ± 1.58) (*t* = 32.264, *p* < 0.001). Correlation analysis revealed a minimal correlation between depression scores and functional status (rho = 0.043, *p* = 0.674) after 3 months, as well as between physical fatigue scores and functional status (rho = –0.168, *p* = 0.094). At the six-month follow-up, a statistically significant correlation was observed between depression scores and functional status (rho = 0.398, *p* < 0.001). Moreover, a strong and significant correlation was identified between physical fatigue scores and functional status (rho = 0.761, *p* < 0.001). Multivariate logistic regression analysis revealed that at three months post-stroke, depression did not significantly affect functional status (odds ratio (OR) = 3.328, *p* = 0.079). However, at six months post-stroke, depression demonstrated a statistically significant effect (OR = 1.436, *p* = 0.030). Physical fatigue showed no significant impact on functional status at three months (OR = 1.010, *p* = 0.927), whereas at six months, it showed a statistically significant effect (OR = 1.581, *p* < 0.001).

**Conclusions:**

These findings underscore the critical importance of integrated care models and early intervention strategies addressing post-stroke depression and physical fatigue to optimize functional outcomes and enhance the overall quality of life for stroke survivors.

## Introduction

Stroke is a principal cause of disability worldwide [1]. The aftermath of a stroke can precipitate a diverse array of physical, cognitive, and emotional challenges, significantly impacting an individual’s functional capacity and health-related quality of life [2, 3, 4]. Within the complex spectrum of post-stroke complications, depression and physical fatigue have emerged as prominent conditions that exert a profound influence on the recovery and rehabilitation of stroke survivors.

Post-stroke depression is a prevalent and consequential complication that has garnered substantial attention in stroke research and clinical practice [5]. A study has indicated that approximately one-third of stroke survivors experience symptoms of depression, highlighting the pervasive nature of this neuropsychiatric sequelae in the post-stroke period [6]. The intricate relationship between post-stroke depression and functional status underscores the multifaceted impact of depressive symptoms on various domains of recovery, including physical rehabilitation, activities of daily living, and social reintegration [7]. Furthermore, post-stroke depression has been implicated in exacerbating cognitive impairments, hindering rehabilitation engagement, and increasing the risk of recurrent vascular events, underscoring its far-reaching implications for stroke survivors [8].

In parallel, post-stroke fatigue has emerged as a significant and underrecognized concern in the stroke survivor population, exerting substantial influence on functional recovery and overall well-being. Post-stroke fatigue is characterized by persistent and debilitating tiredness, diminished energy levels, and an overwhelming sense of exhaustion that transcends normal recuperative mechanisms [9]. The prevalence of post-stroke fatigue has been reported to be 48%, highlighting its widespread impact on individuals in the post-stroke period [10]. The robust association between physical fatigue and functional outcomes has been consistently demonstrated in a previous study, which have shown the detrimental effects of fatigue on mobility, activities of daily living, and participation in rehabilitation programs among stroke survivors [11]. The pervasive influence of physical fatigue on functional status underscores the critical need to investigate its complex interplay with other post-stroke sequelae, such as depression, cognitive impairment, and physical disability, in determining the trajectory of recovery.

The multifactorial nature of post-stroke recovery emphasizes the intricate interplay of biological, psychological, and social factors that collectively underpin the functional outcomes of stroke survivors [12]. The factors synergistically influence various domains of recovery, including physical rehabilitation, emotional well-being, and social reintegration, necessitating a comprehensive understanding of their contributions to overall functional outcomes. Elucidating the distinct and interrelated effects of post-stroke depression and physical fatigue on functional status is essential for developing comprehensive and personalized rehabilitation strategies that address the multidimensional needs of stroke survivors. This study aims to systematically investigate the impact of post-stroke depression and physical fatigue on the functional status of stroke survivors.

## Materials and Methods

### Study Population

All participants gave written informed consent before enrollment, and the signed consent forms were securely archived. This study was approved by the Ethics Review Committee and Ethics Committee of Taihe County People’s Hospital and is under the principles outlined in the Declaration of Helsinki.

### Inclusion and Exclusion Criteria

Inclusion criteria were as follows: patients who had experienced stroke for more than 90 days (since first onset) without recurrent stroke within half a year, age over 18 years, complete clinical data available, ability to understand and cooperate with questionnaire completion, completion of one-year follow-up.

Exclusion criteria were as follows: pre-existing depression or cognitive impairment, severe stroke (impaired consciousness or significant neurological deficits), aphasia or dysarthria leading to the failure of assessment for depression and shame, history of Cushing’s syndrome, adrenal cortical hyperplasia, or tumors, history of hepatitis, tuberculosis, or other infectious diseases, central nervous system infections, dementia, schizophrenia, or other psychiatric disorders.

### Grouping Method

This study conducted a retrospective analysis of clinical data from patients diagnosed with stroke who were admitted to Taihe County People’s Hospital between January 2022 and May 2023. Patients were categorized into two groups based on their prognostic functional status: the good functional status group and the poor functional status group.

In this study, we utilized the Fugl-Meyer Assessment (FMA) to evaluate the functional status of the patients upon admission [13]. The FMA is a comprehensive assessment tool that examines single-joint movements, multi-joint movements, coordination, finger dexterity and speed, ataxia, and reflex activity. The maximum attainable score for the upper extremities is 66, and for the lower extremities is 34, yielding a total possible score of 100. Higher scores indicate less impairment. For this study, we established a custom classification system: a total score exceeding 60 was considered indicative of favorable functional status, while a score below 60 signified unfavorable functional status. The Cronbach’s alpha coefficient for the FMA has been reported as 0.9. The study cohort (N = 100) was stratified into two groups based on their functional status scores: good functional status group (n = 50) (subjects who achieved a score of more than 60 points on the functional assessment test) and poor functional status group (n = 50) (subjects who obtained a score of less than 60 points on the functional assessment test).

### Assessment of Stroke Severity

The National Institutes of Health Stroke Scale (NIHSS) was utilized to evaluate the severity of stroke in both patient cohorts [14]. This scale comprises 11 items, including level of consciousness, facial palsy, visual field impairment, ataxia, and upper and lower limb movement. The NIHSS yields a total score ranging from 0 to 42 points, with higher scores indicating greater neurological impairment. Stroke severity is categorized as follows based on NIHSS scores: 0–1 (normal), 1–4 (mild stroke), 5–15 (moderate stroke), 16–20 (moderate to severe stroke), and 21–42 (severe stroke). Each one-point increase in NIHSS score is associated with a 17% decrease in the probability of a favorable prognosis. The Cronbach alpha coefficient for NIHSS was 0.92.

### Assessment of Depression

The Patient Health Questionnaire-9 (PHQ-9) was employed to assess the symptomatology and clinical status of the patients [15]. The PHQ-9 is a validated self-assessment scale consisting of 9 questions, each covering different symptoms of depression. Patients are required to select the answer that most accurately reflects their symptoms. Each answer corresponds to different scores, with a total score range of 0–27, categorizing the severity of depression into five levels: no depression (0–4), mild depression (5–9), moderate depression (10–14), moderately severe depression (15–19), and severe depression (20–27). The Cronbach alpha coefficient for PHQ-9 was 0.78.

The PHQ-9 was selected as the depression assessment tool in this study due to its concise nature and reduced respondent burden. In contrast to the widely utilized Hamilton Depression Rating Scale (HDRS), which comprises 24 questions, the PHQ-9 consists of only 9 questions with a simplified response format. Considering the physical condition of stroke patients, the PHQ-9 questionnaire with fewer questions was selected for depression score in this study.

### Physical Fatigue Score

The severity of fatigue experienced by stroke patients was assessed using the Fatigue Severity Scale (FSS), which comprises 9 items [16]. The FSS employs a scoring system wherein a total score of <6 indicates the absence of clinically significant fatigue, while a score of ≥36 suggests a heightened propensity for fatigue. Higher scores correlate with increased severity of daily fatigue. The Cronbach alpha coefficient for FSS was 0.96.

### Data Collection

Demographic data, stroke severity indices, depression scale scores, and quantitative measures of physical fatigue scores were collected from the patients’ medical records for analysis.

### Statistical Analysis

Continuous variables (age, body mass index (BMI), depression scores, and physical fatigue scores) were reported as mean ± standard deviation (SD). Categorical variables (gender, hypertension, diabetes, smoking history, drinking history, ischemic stroke incidence, stroke severity, previous stroke history, lesion location, and family history of depression) were presented as frequencies and percentages. Comparisons of demographic characteristics between the two functional status groups were performed using independent samples *t*-tests for continuous variables and chi-squared tests for categorical variables.

The primary objective of this study was to elucidate the differences between groups rather than temporal variations. Consequently, independent samples *t*-tests were employed to assess the differences in depression scores and physical fatigue scores across the two functional status groups at different post-stroke time points (3 months and 6 months). To assess the relationship between patient prognosis and the two aforementioned scores at three- and six-month post-stroke, Spearman’s rank correlation analysis was utilized. This non-parametric measure was selected to examine the strength and direction of the association between these variables.

All statistical analyses were conducted using SPSS 29.0 (SPSS Inc, Chicago, IL, USA). A two-tailed alpha level of 0.05 was established as the threshold for statistical significance across all tests. Results are presented with descriptive statistics, test statistics, and *p*-values to accurately convey the findings.

Variables demonstrating statistically significant differences in both univariate and correlation analysis were subsequently included as covariates in a logistic regression model.

## Results

### Demographic Characteristics

Demographic characteristics were compared between the two functional status groups using independent samples *t*-tests for continuous variables and chi-squared tests for categorical variables. The mean age of participants with good functional status was 65.25 ± 7.12 years, while for those with poor functional status, it was 67.48 ± 6.93 years. No statistically significant difference in age was observed between the two groups (*p* > 0.05). Gender distribution was similar between the two groups, with 28 males and 22 females in the good functional status group, and 25 males and 25 females in the poor functional status group (*p* > 0.05). Furthermore, no significant differences were observed in body mass index (BMI), education level, prevalence of hypertension, diabetes, smoking history, alcohol consumption, ischemic stroke incidence, or stroke severity between the two groups (*p* > 0.05). See Table 1.

**Table 1.** **Demographic characteristics of participants based on functional status**.

Parameter		Good functional status (n = 50)	Poor functional status (n = 50)	*t*/χ^2^	*p*
Age (years)		65.25 ± 7.12	67.48 ± 6.93	1.587	0.116
Gender (M/F)		28/22	25/25	0.361	0.548
BMI (kg/m^2^)		25.75 ± 3.21	25.51 ± 4.05	0.328	0.743
Education situation				0.421	0.517
	Junior high school and below	33 (66%)	36 (72%)		
	Junior high school or above	17 (34%)	14 (28%)		
Hypertension (%)		19 (38%)	17 (34%)	0.174	0.677
Diabetes (%)		11 (22%)	14 (28%)	0.480	0.488
Smoking history (%)		10 (20%)	12 (24%)	0.233	0.629
Drinking history (%)		11 (22%)	13 (26%)	0.219	0.640
Ischemic stroke (Y/N)		35 (70%)	33 (66%)	0.184	0.668
Stroke severity (NIHSS score)		5.24 ± 1.33	5.39 ± 2.14	0.421	0.674
Previous stroke history (%)		2 (4%)	4 (8%)	0.177	0.674
Lesion location (cortical/subcortical)		30 (60%)	28 (56%)	0.164	0.685
Family history of depression (Y/N)		2 (4%)	3 (6%)	0.000	1.000

Abbreviations: M/F, male/female; Y/N, yes/no; BMI, body mass index; NIHSS, National Institutes of Health Stroke Scale.

### Depression Scores (PHQ-9 Score)

At the three-month post-stroke assessment, no statistically significant difference in depression scores was observed between the group with good functional status (3.53 ± 0.84) and the group with poor functional status (3.64 ± 0.75) (*t* = 0.691, *p* = 0.491) (Table 2). However, at the six-month follow-up, a notable disparity emerged. The group exhibiting poor functional status demonstrated markedly elevated depression scores (10.58 ± 3.82) compared to the group with good functional status (7.81 ± 2.12) (*t* = 4.482, *p* < 0.001). See Table 2.

**Table 2.** **Comparison of depression scores (PHQ-9 Score) between the two groups**.

Depression scores	Good functional status (n = 50)	Poor functional status (n = 50)	*t*	*p*
Baseline score	2.57 ± 0.57	2.76 ± 0.59	1.638	0.105
3 months after stroke	3.53 ± 0.84	3.64 ± 0.75	0.691	0.491
6 months after stroke	7.81 ± 2.12	10.58 ± 3.82	4.482	<0.001

Abbreviations: PHQ-9, Patient Health Questionnaire-9.

### Physical Fatigue Scores

At the 3-month post-stroke assessment, no statistically significant difference in physical fatigue scores between the group with good functional status (31.27 ± 3.13) and the group with poor functional status (30.26 ± 5.11) (*t* = 1.188, *p* = 0.238) (Table 3). However, at the 6-month post-stroke evaluation, a significant disparity emerged. The group exhibiting poor functional status demonstrated substantially higher physical fatigue scores (56.87 ± 2.53) compared to the group with good functional status (43.26 ± 1.58) (*t* = 32.264, *p* < 0.001). See Table 3.

**Table 3.** **Comparison of physical fatigue scores by functional status (FSS score) between the two groups**.

Physical fatigue scores	Good functional status (n = 50)	Poor functional status (n = 50)	*t*	*p*
Baseline score	25.27 ± 4.12	25.59 ± 4.14	0.387	0.699
3 months after stroke	31.27 ± 3.13	30.26 ± 5.11	1.188	0.238
6 months after stroke	43.26 ± 1.58	56.87 ± 2.53	32.264	<0.001

Abbreviations: FSS, Fatigue Severity Scale.

### Correlation Analysis

Correlational analysis examining post-stroke depression, physical fatigue, and functional status revealed notable associations (Table 4). At three months post-stroke, minimal and statistically non-significant correlations were observed between depression scores and functional status (rho = 0.043, *p* = 0.674), as well as between physical fatigue scores and functional status (rho = –0.168, *p* = 0.094). In contrast, at six months post-stroke, a moderate and statistically significant correlation emerged between depression scores and functional status (rho = 0.398, *p* < 0.001). Additionally, a strong and significant correlation was identified between physical fatigue scores and functional status (rho = 0.761, *p* < 0.001) at this time point. See Table 4.

**Table 4.** **Correlation analysis between post-stroke depression, physical fatigue, and functional status**.

Index	rho	*p*
3 months after stroke depression scores	0.043	0.674
6 months after stroke depression scores	0.398	<0.001
3 months after stroke FSS score	–0.168	0.094
6 months after stroke FSS score	0.761	<0.001

### Multivariate Logistic Regression Analysis

Based on the multivariate logistic regression analysis examining the relationship between post-stroke depression, physical fatigue, and functional status in stroke survivors, it was observed that at three months post-stroke, depression scores demonstrated an odds ratio (OR) of 3.328 (95% confidence interval (CI): 0.871–12.716), indicating no statistically significant impact on functional status (*p* = 0.079). In contrast, at 6 months post-stroke, depression scores showed an odds ratio of 1.436 (95% CI: 1.035–1.993), suggesting a notable influence on functional status (*p* = 0.030). Regarding physical fatigue, analysis of the 3-month post-stroke assessment revealed that FSS scores had an odds ratio of 1.010 (95% CI: 0.823–1.238), indicating no statistically significant association with functional status (*p* = 0.927). In contrast, the 6-month evaluation demonstrated that FSS scores demonstrated an odds ratio of 1.581 (95% CI: 1.323–1.890), exhibiting a statistically significant impact on functional status (*p* < 0.001). These findings suggest a temporal variation in the influence of post-stroke depression and physical fatigue on the functional outcomes of stroke survivors at different time points post-stroke. See Table 5.

**Table 5.** **Multivariate logistic regression analysis between post-stroke depression, physical fatigue, and functional status**.

Index	OR	OR 95% CI	B	Std error	Wald chi-square	*p*-value
3 months after stroke depression scores	3.328	0.871–12.716	1.202	0.684	3.089	0.079
6 months after stroke depression scores	1.436	1.035–1.993	0.362	0.167	4.683	0.030
3 months after stroke FSS score	1.010	0.823–1.238	0.010	0.104	0.008	0.927
6 months after stroke FSS score	1.581	1.323–1.890	0.458	0.091	25.336	<0.001

Abbreviations: OR, odds ratio; CI, confidence interval; B, bate; Std error, standard error.

## Discussion

The impact of post-stroke depression and physical fatigue on functional status represents a critical aspect of stroke recovery that warrants thorough investigation. This study aimed to explore the relationships between post-stroke depression, physical fatigue, and functional status at different time points following stroke. The findings provide insights into the dynamic nature of these factors and their influence on functional outcomes in stroke patients.

Post-stroke depression is a well-recognized phenomenon that can significantly affect the overall recovery and functional status of stroke patients [17]. The NIHSS scores in our study indicated that the majority of participants presented with moderate stroke severity, while a minority exhibited mild stroke symptoms. This distribution aligns with our inclusion and exclusion criteria, minimizing potential confounding effects on the study results. We employed the Patient Health Questionnaire-9 (PHQ-9) to assess depression severity at three and six months post-stroke. Our analysis revealed no statistically significant difference in depression scores between patients with good and poor functional status at the 3-month follow-up. However, a notable disparity emerged at the 6-month assessment point. These findings underscore the potential for post-stroke depression to manifest or intensify over time, with a particularly pronounced impact on functional status among patients with poor prognostic functional outcomes. The observed disparity in depression scores between the two groups at six months post-stroke highlights the need for continuous monitoring of mental health status beyond the acute phase of stroke, as the onset of depression may occur or escalate in the subacute or chronic phases, thereby potentially influencing functional recovery.

Our findings demonstrated a significant correlation between depression scores and functional status at six months post-stroke. This association suggests that post-stroke depression may contribute to poorer functional outcomes, consistent with previous literature documenting the detrimental effects of depression on rehabilitation and recovery following stroke [18]. Early identification and intervention for post-stroke depression may therefore be crucial for improving functional status and overall quality of life in stroke survivors.

Our study elucidated the significance of physical fatigue, alongside post-stroke depression, as a crucial determinant of functional status in stroke patients. Significantly elevated physical fatigue scores were observed in the group exhibiting poor functional status at six months post-stroke, indicating a robust association between physical fatigue and long-term functional outcomes. These findings underscore the necessity of recognizing and addressing physical fatigue as a salient factor in stroke recovery. The strong and statistically significant correlation between physical fatigue scores and functional status at six months post-stroke emphasized the profound impact of physical fatigue on functional recovery. Physical fatigue is a multifaceted symptom encompassing both central and peripheral components. It manifests as a profound sense of exhaustion, decreased endurance, and heightened perceived effort during physical activities [19]. The impact of physical fatigue on functional status may be observed through its effects on mobility, participation in rehabilitation programs, and overall engagement in activities of daily living. The intricate relationship between physical fatigue and functional status necessitates the development and implementation of tailored interventions that address fatigue management as an integral component of stroke rehabilitation.

The observed associations between depression, physical fatigue, and functional status underscore the intricate relationship among these factors and their collective influence on the rehabilitation and recovery trajectory of stroke survivors. These findings have significant clinical implications, emphasizing the need for a comprehensive, multidimensional approach to post-stroke care that addresses not only physical impairments but also psychological and emotional well-being. Implementing effective interventions targeting post-stroke depression and physical fatigue management could be pivotal for optimizing functional outcomes and enhancing the overall recovery process in stroke survivors.

The significant impact of post-stroke depression and physical fatigue on the functional status of stroke survivors can be attributed to biological, psychological, and social factors [20]. Elucidating the mechanisms underlying the significant impact of these factors on functional outcomes is crucial for developing targeted interventions and comprehensive care strategies for stroke survivors. The neurobiological effects of stroke, including neuroinflammatory processes, may contribute to the onset and progression of post-stroke depression and physical fatigue. Stroke-induced neuroinflammation, along with alterations in neurotransmitter systems—particularly those involving serotonin, dopamine, and norepinephrine—has been implicated in the pathophysiology of post-stroke depression [21]. Dysregulation of these neurochemical pathways can lead to mood disturbances, anhedonia, and reduced motivation, profoundly impacting functional status and impeding rehabilitation progress. The neuroinflammatory response following a stroke can contribute to the manifestation of physical fatigue through multiple mechanisms. These include cytokine-mediated sickness behavior, disruption in cellular energy metabolism, and alterations in neural circuits regulating fatigue perception. The complex neurobiological sequelae underlying these processes highlight the intricate interplay between the neurovascular consequences of stroke and the subsequent development of post-stroke depression and physical fatigue. Both of these phenomena exert a significant influence on functional status in stroke survivors [22]. Stroke-induced physical impairments, including motor deficits, gait disturbances, and limitations in activities of daily living (ADL), can engender feelings of helplessness, frustration, and social isolation, thereby increasing susceptibility to depressive symptomatology and physical fatigue [23]. Conversely, the presence of post-stroke depression and physical fatigue may exacerbate functional limitations by diminishing motivation, reducing engagement in rehabilitation activities, and impeding overall physical recovery [24]. The bidirectional interaction between physical disability and psychological symptom creates a challenging cycle that impedes functional improvement, underscoring the importance of addressing both the physiological and psychological aspects of post-stroke rehabilitation. Furthermore, the psychosocial consequences of stroke and its sequelae play a pivotal role in determining the course of post-stroke depression, physical fatigue, and functional outcomes. People with stroke develop specific dysfunctions that prevent them from managing simple personal care tasks, engaging in valued activities, and fulfilling important life roles, undermining their sense of self, leading to reduced self-worth and changes in self-image and identity [25]. These psychosocial stressors, when combined with persistent physical disability and cognitive impairments, may contribute to the development of depressive symptoms and physical fatigue, which can impede functional recovery [26].

The significant impact of post-stroke depression and physical fatigue on functional status in stroke survivors stems from the complex, multifactorial nature of stroke recovery, which encompasses interrelated neurobiological, psychosocial, and cognitive factors [27, 28, 29]. Addressing the underlying factors requires a comprehensive and integrated approach to post-stroke care, incorporating neurorehabilitation, psychosocial support, cognitive interventions, and interdisciplinary collaboration. By recognizing and targeting the multifaceted nature of post-stroke depression and physical fatigue, healthcare providers can optimize functional outcomes and enhance the overall quality of life for individuals affected by stroke.

This study had several limitations that warrant consideration. First, the retrospective design of the study constrained our capacity to prospectively collect comprehensive data and establish causal relationships among the variables of interest. Additionally, the relatively small sample size and single-center approach may potentially impact the generalizability of our findings. The study population predominantly comprised individuals with moderate stroke severity, with a limited representation of mild stroke cases. Consequently, the generalizability of our conclusions is restricted to this specific subpopulation. Future research endeavors should aim to address these limitations through the implementation of large-scale, multi-center prospective cohort studies. These studies should comprehensively evaluate the multifaceted factors impacting functional outcomes in post-stroke patients. Moreover, the inclusion and exclusion criteria utilized in this study may have introduced selection bias, potentially limiting the generalizability of our findings. The utilization of de-identified patient data limited our ability to explore additional relevant variables that could potentially confound the observed associations.

## Conclusions

In conclusion, this study sheds light on the intricate interplay among post-stroke depression, physical fatigue, and functional status in stroke survivors. The observed associations between these factors underline the necessity of implementing a comprehensive, patient-centered approach to post-stroke care that addresses the multifaceted needs of stroke survivors.

## Availability of Data and Materials

The datasets used and/or analyzed during the current study were available from the corresponding author on reasonable request.
